# Li_3_InCl_6_ as an Active Filler Improves Interfacial Stability of PEO-LiTFSI Composite Solid Polymer Electrolytes for All-Solid-State Lithium Metal Batteries

**DOI:** 10.1021/acsomega.6c02071

**Published:** 2026-08-20

**Authors:** Hongrui Kang, Weiye Ma, Jan P. Hofmann, Zhonghui Gao

**Affiliations:** † School of Materials Science and Engineering, 12605 Tianjin University, Tianjin 300350, P. R. China; ‡ Surface Science Laboratory, Department of Materials- and Geosciences, 26536 Technical University of Darmstadt, Peter-Grünberg-Straße 4, 64287 Darmstadt, Germany

## Abstract

Composite solid polymer electrolytes (CSPEs) with inorganic fillers are of great interest for advanced all-solid-state lithium–metal batteries (ASSLMBs), enabling high energy density and enhanced safety. Despite increasing interest, the effects of different inorganic fillers on the interface between CSPEs and the lithium metal anode (LMA) remain not fully understood. Herein, we systematically study the contact behavior between the LMA and CSPEs containing PEO, LiTFSI, as well as oxide- (Li_6.4_La_3_Zr_1.4_Ta_0.6_O_12_), sulfide- (Li_6_PS_5_Cl), and halide-based (Li_3_InCl_6_) fillers by evaporating lithium metal stepwise onto CSPE thin films under ultrahigh vacuum conditions, followed by *in-system* X-ray photoelectron spectroscopy (XPS) analysis. XPS results demonstrate that halide-based fillers can facilitate the formation of electron-rich Li–O and Li–S species at the interfaces, acting as a passivation layer, which can prevent further decomposition of the CSPE and thus improve interfacial stability. In contrast, the incorporation of oxide- and sulfide-based fillers does not promote the interfacial reactions. Impedance spectroscopy as a function of resting time also shows that the incorporation of a halide-based filler reduces the CSPE/LMA interfacial impedance, which is beneficial for increasing the critical current density for lithium dendrite formation. The incorporation of active inorganic fillers to induce protective interphases offers a novel strategy for interfacial engineering of ASSLMBs.

## Graphical abstract

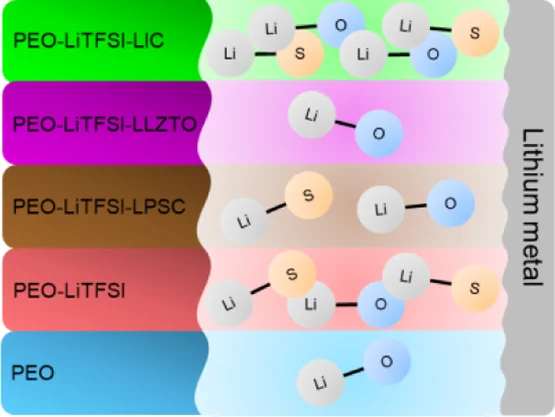

## Introduction

With the increasing demand for higher kilometer range of electric vehicles, higher energy density lithium-ion batteries (LIBs) are required.^1,2^ After more than 30 years of development, the practical energy density of conventional LIBs with organic liquid electrolytes, polymer separators, and graphite anodes (with a capacity of only 372 mAh g^–1^) has already reached its limit (∼300 Wh kg^–1^). In order to achieve further enhancement, lithium metal anodes (LMAs) with an extremely high specific capacity of 3860 mAh g^–1^ and the lowest reduction potential of −3.04 V versus the standard hydrogen electrode, are considered to be an excellent anode material.^3–7^ However, the use of lithium metal in conventional LIBs is facing critical issues, the most severe of which is the growth of lithium dendrites, which could pierce through the separator and result in an internal short circuit, followed by thermal runaway and even fire/explosion.^8,9^ Also the intrinsic, highly reactive nature of the LMA makes it a strong reducing agent, leading to reductive degradation reactions, followed by the decomposition of electrolytes at the contact as well as the formation of a solid electrolyte interphase (SEI). The nature and characteristics of the SEI are crucial to the performance of LIBs because its formation is one of the causes of irreversible capacity loss.^10–13^

By replacing liquid electrolytes and polymer separators with electrochemically, thermally, and mechanically stable solid electrolytes, which are able to suppress uneven electrodeposition of lithium and thus hinder the formation of lithium dendrites, all-solid-state lithium metal batteries (ASSLMBs) are expected to be next-generation battery systems.^14–18^

SEs can be grouped into three categories: inorganic solid electrolytes (ISEs), solid polymer electrolytes (SPEs), and composite solid polymer electrolytes (CSPEs).^19,20^ ISEs exhibit high ionic conductivity (10^–4^–10^–2^ S cm^–1^) and high lithium-ion transfer numbers (close to 1) at room temperature (RT). These materials include oxide-, sulfide- and halide-based SEs.^21–23^ For oxide-based SEs, perovskite-type (e.g., Li_0.34_La_0.56_TiO_3_), garnet-type (e.g., Li_7_La_3_Zr_2_O_12_), and NASICON-type (e.g., Li_1.3_Al_0.3_Ti_1.7_(PO_4_)_3_) are the most representative ones, with ionic conductivities in the range of 10^–5^–10^–3^ S cm^–1^, possessing good thermal stability and high Young’s moduli (>150 GPa). However, due to their high mechanical rigidity, it is often difficult to integrate them into ASSLBs.^24,25^ Typical sulfide-based SEs are Li_7_P_3_S_11_, Li_10_GeP_2_S_12_, Li_2_S–P_2_S_5_, and Li_6_PS_5_Cl, although they have higher ionic conductivities (up to 10^–2^ S cm^–1^) than oxide-based SEs, and also good mechanical flexibility, their chemical stability is poor with being especially sensitive to moisture.^26–28^ Halide-based SEs with the general composition of Li_3_MX_6_ (M^3+^, X = Cl, Br, I) have drawn more attention, because the Coulombic forces between lithium ions and monovalent halogens are weaker in comparison to divalent sulfur or oxygen ions. Nevertheless, their instability and incompatibility against LMA still remain as a challenge.^29,30^ In contrast, SPEs consist of a polymer matrix (e.g., poly­(ethylene oxide) (PEO), polypropylene carbonate (PPC), polyacrylonitrile (PAN), or polyethylene carbonate (PEC)) and lithium salts (e.g., LiC_2_F_6_NO_4_S_2_ (LiTFSI), LiSO_3_CF_3_, or LiClO_4_),^31–33^ and typically exhibit good flexibility, processing properties, and electrode wettability but also show low ionic conductivity (<10^–5^ S cm^–1^) at RT due to the crystallization of the polymer.^34,35^

One promising strategy to enhance the performance of SPEs is incorporating active ISE fillers to form CSPEs with significantly improved ionic conductivity, electrochemical stability, and mechanical strength.^19,36–38^ However, oxide-based SE fillers are inherently brittle, and they often have poor interfacial contact with the polymer matrices, leading to reduced Li^+^ transport across the filler–polymer interface. Adding them in significant amounts into SPEs also reduces the flexibility and processability.^39,40^ Since sulfide-based SE fillers are highly sensitive to moisture and can form toxic H_2_S gas when in contact with water, incorporating them into SPEs often requires careful processing under an inert atmosphere, which increases production costs.^41^ In comparison, halide-based SE fillers exhibit high intrinsic ionic conductivities (10^–3^–10^–2^ S cm^–1^); when incorporated into polymer matrices, they can enhance the overall ionic conductivity of the CSPEs. Among them, Li_3_InCl_6_ and Li_3_YCl_6_ have been frequently investigated due to their wider electrochemical stability windows (2.38–4.26 V and 0.62–4.02 V versus Li^+^/Li, respectively) and better chemical stability toward high-voltage cathode materials.^30,42^

X-ray photoelectron spectroscopy (XPS) has emerged as a powerful tool for probing interfacial chemistry, enabling direct observation of chemical evolution and interphase formation at the buried CSPE/LMA interface, thereby providing a fundamental understanding of interfacial reaction mechanisms. To our knowledge, no experimental studies on the interface properties of CSPE with halide-based SE filler against LMA have been reported so far. Since solid-state interfaces are typically embedded, precise interfacial design and reliable characterization are key to achieving stable performance.^43,44^ Herein, interfaces between CSPE with halide- (Li_3_InCl_6_, LIC), oxide- (Li_6.4_La_3_Zr_1.4_Ta_0.6_O_12_, LLZTO), and sulfide (Li_6_PS_5_Cl, LPSC)-based SE fillers and LMA are investigated by an *in-system* approach with XPS. The ultralow impedance of the SEI is demonstrated by impedance spectroscopy as a function of resting time and cycling, performed on symmetric cells with lithium metal electrodes. Our study reveals distinct interfacial behaviors induced by the different SE fillers. Importantly, we report a novel interphase formation mechanism promoted by halide-based SE fillers, which effectively improves interfacial stability and suppresses lithium dendrite growth.

## Experimental Methods

### Material Synthesis

As precursor materials for CSPE samples, poly­(ethylene oxide) (H­(OCH_2_CH_2_)_n_OH, PEO, Aladdin Scientific, average Mv 300,000) with a mass of 500 mg and bis­(trifluoromethane)­sulfonimide lithium salt (CF_3_SO_2_NLiSO_2_CF_3_, LiTFSI, Aladdin Scientific, 99%) were premixed in the molar ratio of [EO]:[Li] = 15:1. Then, lithium indium chloride (Li_3_InCl_6_, MTI Corp, 99%), accounting for 10, 15, 20, and 30 wt % of the total amount of the mixture, was added into the premixture, respectively, for the preparation of the PEO-LiTFSI-LIC samples. In addition, both tantalum-doped lithium lanthanum zirconium oxide (Li_6.4_La_3_Zr_1.4_Ta_0.6_O_12_, LLZTO, PI-KEM, 99%) and lithium phosphorus sulfur chloride (Li_6_PS_5_Cl, LPSC, Tianshi Kefeng, 99%), each accounting for 10 wt % of the total amount of the mixture, were used to prepare reference samples.

All mixtures were dissolved in acetonitrile (CH_3_CN, Aladdin Scientific, 99%) with a volume of 5 mL inside an argon-filled glovebox (Super Pro (1220/750/900), Mikrouna). The solution was stirred for 24 h by using a magnetic stirrer (HJ-4, Suri Instruments). Then, the solution was uniformly coated on PTFE plates with a spatula. The plates were transferred to a vacuum drying oven (DZF-6022, Yihang Instruments) and heated to 60 °C for 24 h to ensure that acetonitrile was completely evaporated. After cooling, the dried films were transferred back to the glovebox, peeled off from the plates, and finally cut into round shapes with a diameter of 18 mm for subsequent use.

### Materials Characterization

All pristine CSPE samples were mounted onto sample holders inside an argon-filled glovebox (MB10 Compact, MBraun GmbH) with both H_2_O and O_2_ levels of <0.1 ppm, then transferred under an argon atmosphere to the XPS machine for initial measurements.

XPS data were obtained on a Physical Electronics PHI Versaprobe 5000 spectrometer as part of the DAISY-BAT ultrahigh vacuum cluster tool in fixed analyzer transmission mode, using monochromatic Al Kα radiation (*hν* = 1486.6 eV, spot diameter of 200 μm, and power of 50 W) at an analyzer angle of 45°, with a pass energy of 23.50 eV and a step size of 0.1 eV for region scans. For survey scans over a range of 1350–0 eV, a pass energy of 187.85 eV and a step size of 0.8 eV were used. Due to the low electronic conductivity of all CSPE samples, charge neutralization has been applied using electron flooding. Binding energy (BE) calibration was performed by setting the C 1s signal of sp^3^ carbon to 284.8 eV via CasaXPS software.

Hereafter, lithium metal was evaporated stepwise onto all pristine CSPE samples using a lithium dispenser (LI/NF/1.25/25 FT10 + 10, SAES Getters). The evaporation process was carried out inside an ultrahigh vacuum (UHV) dispenser chamber with a base pressure of 10^–9^ mbar, by applying a current of 7.5 A to the lithium dispensers (see Supporting Information, Figure S8a). Each evaporation lasted for 120 s, followed by one XPS measurement.

After completion of the lithium evaporation sequence, all CSPE samples were transferred to another UHV chamber with a base pressure of 10^–8^ mbar for conducting a heat treatment (HT). At first, argon was introduced into the chamber with a flux of 6 sccm until the pressure reached 8.0 × 10^–3^ mbar. Then, the CSPE samples were heated to 90 °C and kept for 3 h. After HT, the CSPE samples were left inside the chamber to cool down to room temperature, followed by final XPS measurements. During transfer between the XPS machine, dispenser chamber, and anode chamber, UHV conditions were maintained (see Supporting Information, Figure S8b).

### Electrochemical Impedance Spectroscopy Measurements

CR2025-type Li/PEO-LiTFSI-10 wt % LIC/Li symmetric coin cells were prepared by using a button cell sealing machine (MSK-160E, MTI Corp.) with a pressure of 0.7 t. All symmetric cells were further heated to 60 °C for 48 h inside a vacuum drying oven. Electrochemical impedance spectroscopy and cycling characterization were performed on these symmetric cells by using a potentiostat (CT3004A, LANHE).

## Results and Discussion

Figure 1 shows the evolution of O 1s, In 3d, S 2p, and Li 1s spectra of the pristine (P) PEO-LiTFSI-30 wt % LIC sample during stepwise lithium evaporation and following heat treatment (HT). From the O 1s spectra in Figure 1a, it can be seen that the pristine PEO-LiTFSI-30 wt % LIC sample exhibits a strong C–O peak (533.0 eV), contributed by the PEO component, a carbonate peak (531.5 eV), and a weak peak at 534.7 eV, probably caused by residual water adsorption. After the first lithium evaporation of 120 s, the intensity of the C–O peak is suppressed, and the carbonate peak becomes stronger. However, with further lithium evaporation, the C–O peak gradually restores its intensity.

**Figure 1:**
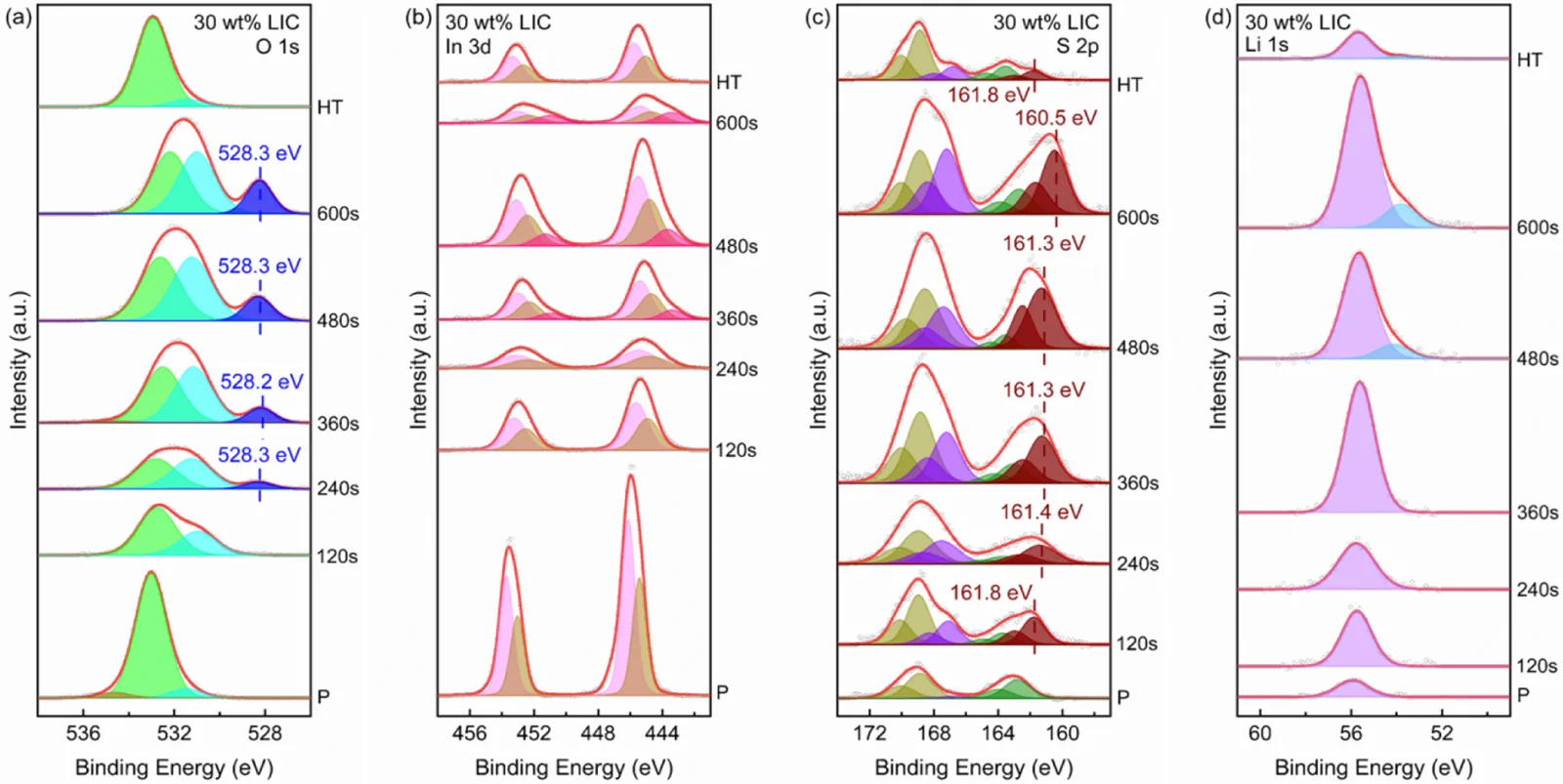
X-ray photoelectron spectra of (a) O 1s, (b) In 3d, (c) S 2p, and (d) Li 1s of the PEO-LiTFSI-30 wt % LIC sample at pristine during stepwise lithium evaporation, and post-HT conditions (HT was conducted after stepwise lithium evaporation of 600 s).

After lithium evaporation of 240 s, a new peak at 528.3 eV emerges and becomes stronger with further lithium evaporation, while disappearing with HT. Given that this peak is located at a relatively low BE position, it can be assigned to an electron-rich Li–O species present at the CSPE surface. We demonstrate that during sample preparation, the structure of LIC collapsed under the influence of acetonitrile and In^3+^ sites were exposed, by measuring X-ray photoelectron spectra of the In 3d core level of LIC powders under different conditions, namely pristine powder, dissolved in acetonitrile then dried in an Ar-filled glovebox (LIC-acetonitrile), and dissolved in acetonitrile with LiTFSI then dried in an Ar-filled glovebox (LiTFSI-LIC-acetonitrile). In comparison with pristine powder (BE (In 3d_5/2_) = 446.2 eV), the LIC signal of LIC-acetonitrile powder starts shifting to a lower BE position (BE (In 3d_5/2_) = 446.1 eV), while for LiTFSI-LIC-acetonitrile powder, the signal shifts significantly lower (BE (In 3d_5/2_) = 446.1 eV), indicating that the coordination environment of LIC is changed with the presence of acetonitrile. Meanwhile, exposed In^3+^ sites tend to absorb TFSI^–^ since adsorption energies of TFSI^–^, CH_3_CN, and 3PEO are −3.31 eV, −1.14 eV, and −1.19 eV, respectively.^45,46^ This preferable absorption of exposed In^3+^ sites with TFSI^–^ enhances the interaction with PEO chains and results in amorphization (see Supporting Information, Figure S1). During stepwise lithium evaporation, electron-rich Li–O species gradually separated out from the amorphous bulk and loosely attached to the CSPE surface, leading to increasingly stronger peak intensities. Eventually, these species left the CSPE surface during HT as an effect of both heat and ultrahigh vacuum.

Regarding the pristine state, i.e., prior to lithium evaporation, strong Li_3_InCl_6_ (BE (In 3d_5/2_) = 446.1 eV) and In_2_S_3_ (BE (In 3d_5/2_) = 445.4 eV) peaks can be seen (Figure 1b). During stepwise lithium evaporation and after HT, the intensities of both peaks decreased. After 360 s of lithium evaporation, metallic indium signals (BE (In 3d_5/2_) = 443.4–443.7 eV) are detected because In^3+^ is reduced by lithium metal. Although Li_3_InCl_6_ and In_2_S_3_ peaks become weaker, there is no more metallic indium peak after HT, meaning that the reduction reaction caused by lithium metal stops. Contrary to the findings of Riegger et al.,^29^ who reported that pure Li_3_InCl_6_ SE is not stable against lithium metal and keeps decomposing during stepwise lithium evaporation, our finding demonstrates that with the help of organic PEO and LiTFSI, the stability of Li_3_InCl_6_ SE toward lithium metal can be significantly improved.

There is evidence for electron-rich Li–S species in the S 2p spectra as well (Figure 1c), which locate at relatively low BE positions. This doublet with BE (S 2p_3/2_) = 160.5–161.8 eV emerges after the first lithium evaporation and becomes stronger with more lithium metal evaporated onto the CSPE sample. Although this peak does not completely disappear after HT, its intensity is significantly lower than before, exhibiting the same trend as the electron-rich Li–O species in the O 1s spectrum, suggesting similar weak bonding to the CSPE surface. Both electron-rich Li–O and Li–S species constitute a passivating SEI at the interface between reactive lithium metal and the CSPE bulk, which prevents further direct contact and the resulting reactions. Next to the electron-rich Li–S species, Li_2_SO_4_ (BE (S 2p_3/2_) = 168.4 eV), Li_2_SO_3_ (BE (S 2p_3/2_) = 166.6 eV), and Li_2_S peaks (BE (S 2p_3/2_) = 162.8 eV) can be detected at the pristine state, and the intensities of the former two species first increase with lithium evaporation, then suddenly decrease after HT, while the intensity of Li_2_S remains almost constant.

As shown in the Li 1s spectra (Figure 1d), the initial Li_2_CO_3_ component (55.9 eV) at the pristine state appears weak and then becomes much stronger with progressing lithium evaporation. However, its intensity drops rapidly after HT. The peak of metallic lithium (54.2 eV) appears after evaporating lithium for 480 s and remains in small amounts after HT. Despite peak intensities in the In 3d spectra decreasing between pristine and after HT conditions, it seems that the impact of evaporated lithium metal on the CSPE can be removed by the heat treatment.

In order to investigate the effect of different LIC percentages in the CSPE, further stepwise lithium evaporations of 600 s and following HT were carried out on CSPE samples with 10, 15, and 20 wt % LIC content. For all PEO-LiTFSI-10 wt % LIC, PEO-LiTFSI-15 wt % LIC, and PEO-LiTFSI-20 wt % LIC samples, peaks of electron-rich Li–O and Li–S species emerge at BE of ∼528 eV (O 1s) and ∼161 eV (S 2p_3/2_), respectively, during evaporation processes (see Supporting Information, Figures S2, S3). As shown in Figure 2a, both impacts of lithium metal on oxygen species of PEO-LiTFSI-10 wt % LIC and PEO-LiTFSI-15 wt % LIC samples are almost completely removed after HT, while peaks of carbonate and electron-rich Li–O species persist in the O 1s spectrum of the PEO-LiTFSI-20 wt % LIC sample. Unlike PEO-LiTFSI-30 wt % LIC sample, the intensities of indium species of PEO-LiTFSI-10 wt % LIC, PEO-LiTFSI-15 wt % LIC, and PEO-LiTFSI-20 wt % LIC samples even increase after HT (Figure 2b), and no trace of metallic indium is found.

**Figure 2:**
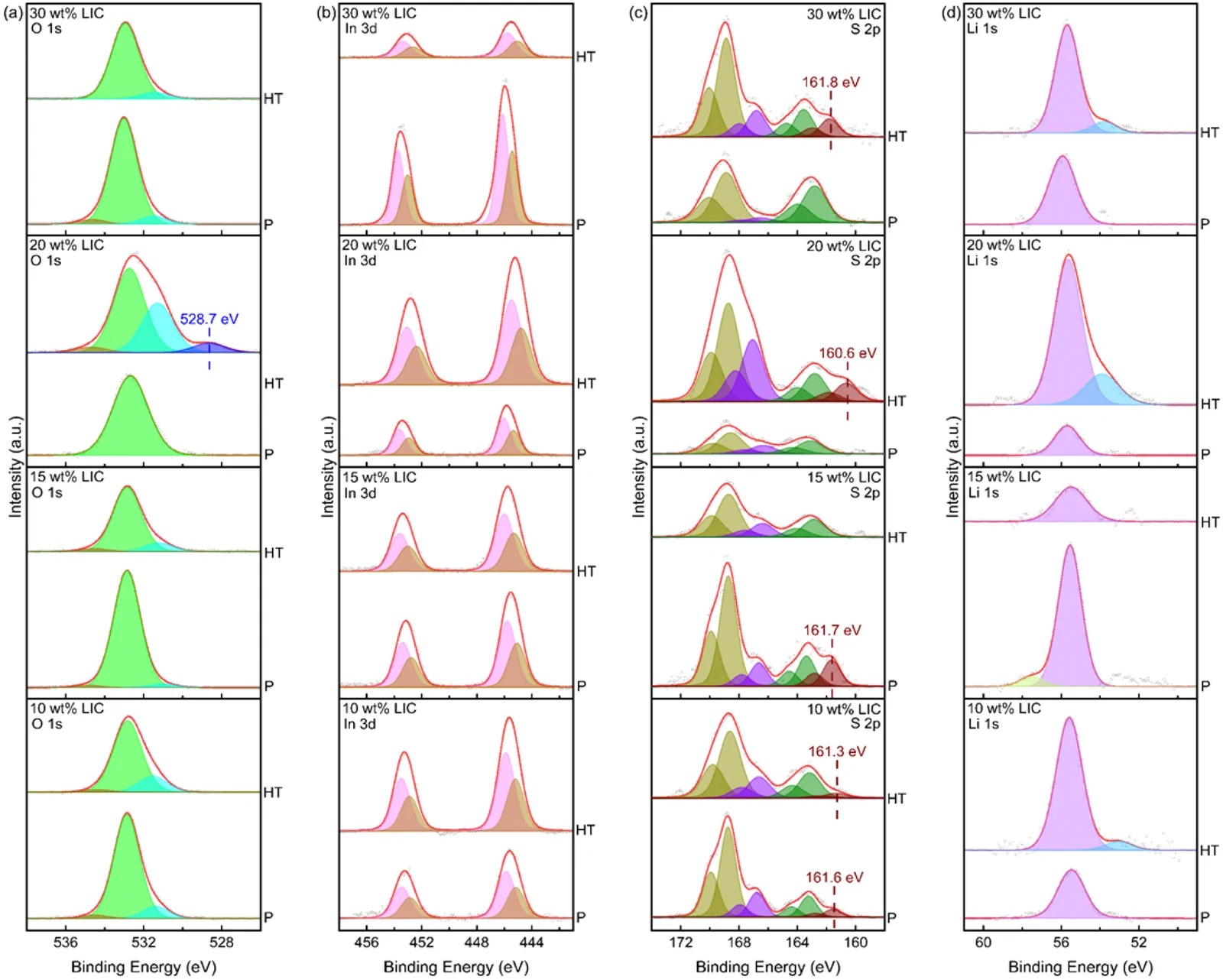
X-ray photoelectron spectra of (a) O 1s, (b) In 3d, (c) S 2p, and (d) Li 1s of CSPE samples with different LIC contents under pristine and post-HT conditions.

Electron-rich Li–S species already exist in CSPE samples with relatively low LIC percentage (Figure 2c), indicating that even without the presence of external lithium metal, Li^+^ from structurally collapsed LIC is able to bond with S^2–^ from LiTFSI. Among all four CSPE samples, the PEO-LiTFSI-20 wt %LIC sample exhibits the most significant inconsistency, which has the strongest Li_2_CO_3_ peak and the most remaining metallic lithium on the surface after HT (Figure 2d), probably caused by the uneven distribution of PEO, LiTFSI, and LIC. In general, all CSPE samples with different LIC percentages are not severely reduced by evaporated lithium metal, showing enhanced stability of LIC. According to Li 1s spectra, it appears that organic components are able to absorb lithium metal without any chemical reactions taking place and release it to evaporate during HT. We note a scatter of intensities of lithium species peaks in Li 1s spectra of PEO-LiTFSI-10 wt % LIC, PEO-LiTFSI-15 wt % LIC, and PEO-LiTFSI-20 wt % LIC (see Supporting Information, Figure S4), which can be probably caused by unevenly distributed absorbed lithium metal in CSPE samples. Another explanation might be that after transferring CSPE samples from the dispenser chamber back to the XPS analysis chamber (see Supporting Information, Figure S8b), the measurement position of the XPS slightly changed, leading to variation of intensities of peaks. In order to investigate this phenomenon, stepwise lithium evaporation was conducted on pure PEO and PEO-LiTFSI reference samples.

Figure 3a shows the evolution of the Li 1s spectra of a pure PEO reference sample during the evaporation process and after HT. After the first lithium evaporation of 120 s, Li_2_O (55.5 eV) and LiOH (54.7 eV) emerge, and their intensities continuously increase until evaporation of 480 s. However, after another evaporation of +120 s, the intensities of both peaks remain almost identical, and an additional peak of metallic lithium at 52.7 eV appears. Subsequent HT removal of the signature of evaporated lithium resulted in significantly weakened peaks. The reason can be explained by the fact that after evaporation of 480 s, a saturation of the amount of lithium metal that can be absorbed by PEO was reached; therefore, extra lithium metal could only attach to the surface of the PEO sample. During HT, lithium metal both inside and attached to PEO was evaporated to vacuum, resulting in a significant decrease in peak intensities.

**Figure 3:**
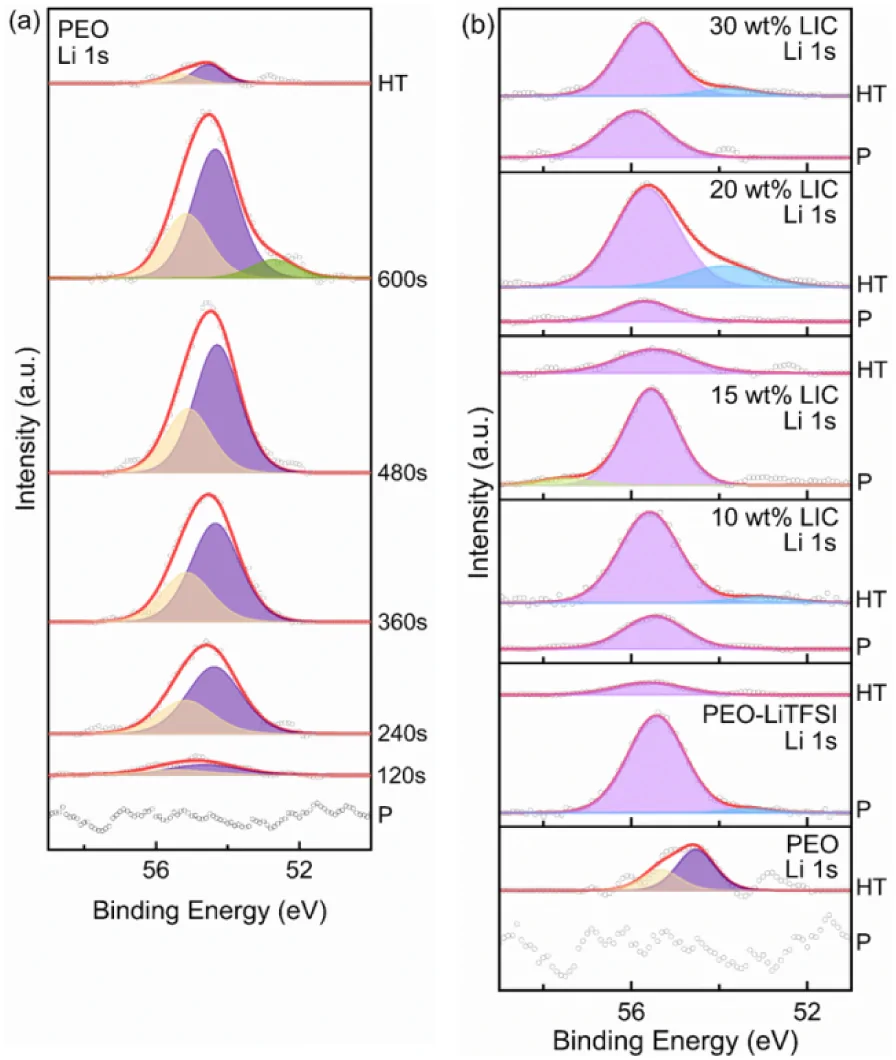
X-ray photoelectron spectra of Li 1s of (a) PEO sample under pristine, during stepwise lithium evaporation, and post-HT conditions, and (b) PEO and PEO-LiTFSI reference samples, as well as four CSPE samples with different LIC contents under pristine and post-HT conditions.

From the comparison of Li 1s spectra of pure PEO and PEO-LiTFSI reference samples, along with four CSPE samples with different LIC contents, obtained under pristine conditions, as well as after stepwise lithium evaporation of 600 s and HT conditions (Figure 3b), we can see that adding LiTFSI can suppress the accumulation of lithium metal, and HT can even reduce the Li signal from LiTFSI itself. For PEO-LiTFSI-LIC samples, except for the one with 15 wt % LIC, the rest exhibit enhanced Li_2_CO_3_ peaks as well as traces of metallic lithium after stepwise lithium evaporation of 600 s and HT.

For the pure PEO sample, a small amount of electron-rich Li–O species formed during the evaporation process. As for the PEO-LiTFSI sample, more can be detected, but still less than the amount formed inside the PEO-LiTFSI-30 wt %LIC sample. Additionally, there is already a trace of electron-rich Li–S species inside the PEO-LiTFSI pristine sample (see Supporting Information, Figures S5 and S6). However, the amount of electron-rich Li–S species remained almost the same during the evaporation process, suggesting that the presence of LiTFSI is only helpful in forming electron-rich Li–O species. Two more CSPE samples with Li_6.4_La_3_Zr_1.4_Ta_0.6_O_12_ (LLZTO) and Li_6_PS_5_Cl (LPSC) fillers were used to further investigate the effect of the chemistry of the inorganic fillers. For the PEO-LiTFSI-LLZTO sample, only a few electron-rich Li–O and Li–S species formed during stepwise lithium evaporation (Figure 4a and 4b). Compared to the PEO-LiTFSI-30 wt %LIC sample, fewer electron-rich Li–O species formed in the case of the PEO-LiTFSI-LPSC sample. Also, fewer electron-rich Li–S species formed during the evaporation process, but their amount significantly increased after HT (Figure 4c and 4d), indicating that HT has a counter-effect when the inorganic filler is LPSC.

**Figure 4:**
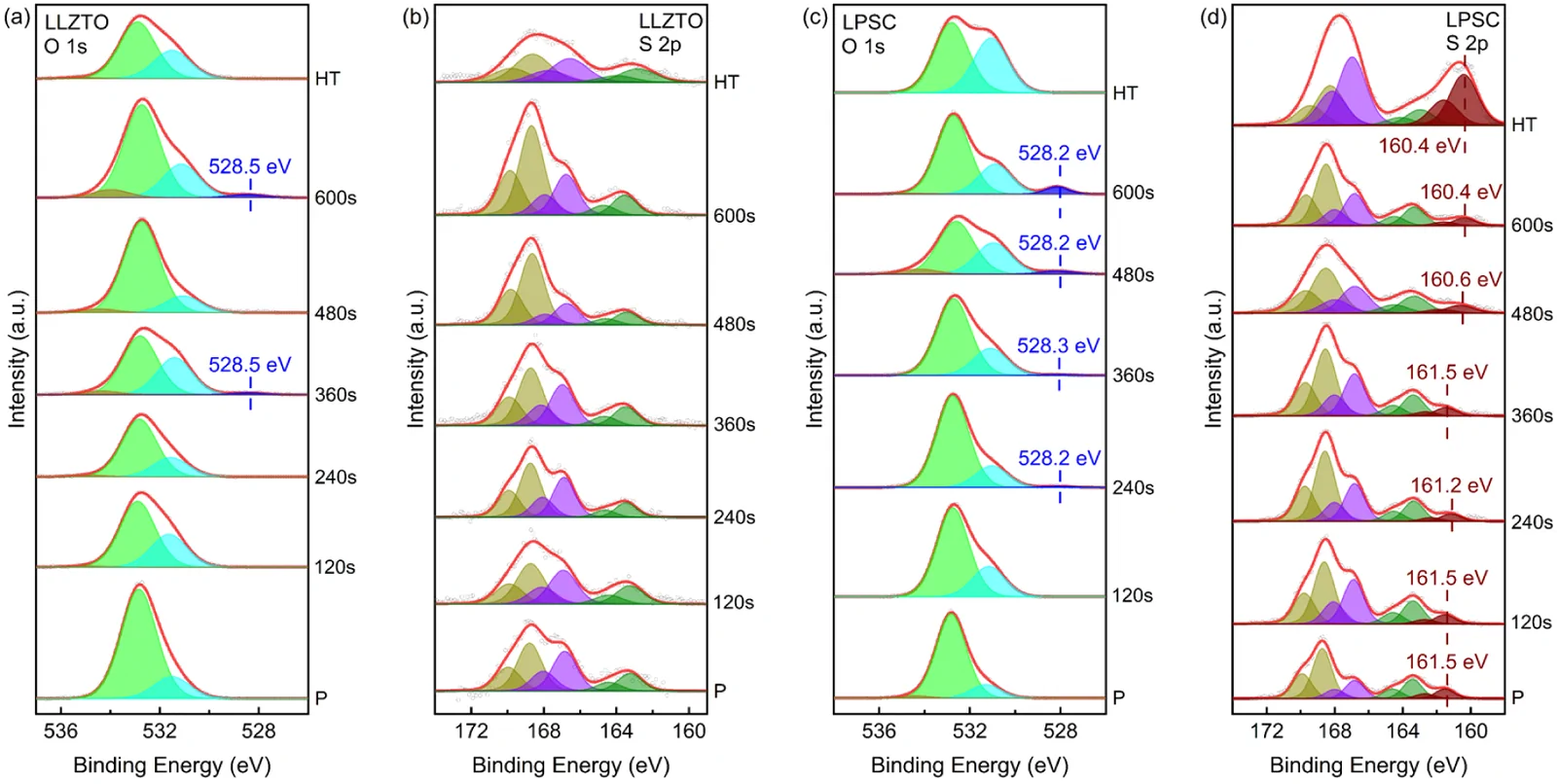
X-ray photoelectron spectra of O 1s of (a) PEO-LiTFSI-LLZTO and (c) PEO-LiTFSI-LPSC samples, and S 2p of (b) PEO-LiTFSI-LLZTO and (d) PEO-LiTFSI-LPSC samples at pristine, during stepwise lithium evaporation, and post-HT conditions.

The atomic concentration (At %) of electron-rich Li–O and Li–S species derived from O 1s and S 2p core levels (i.e., 100% for each core level), formed during stepwise lithium evaporation and remaining at post=HT conditions, can be found in Tables 1 and 2, respectively. Among all CSPE samples with LIC active filler, the PEO-LiTFSI-30 wt % LIC sample exhibits a more regular change in electron-rich Li–O and Li–S species, though the At % of electron-rich Li–S species slightly decreased after a lithium evaporation time of 600 s, probably due to spatial heterogeneity in the range of 20–50 μm (see Supporting Information, Figure S7). For PEO-LiTFSI-10 wt % LIC and PEO-LiTFSI-20 wt % LIC samples, large amounts of electron-rich Li–O and Li–S species are formed after a lithium evaporation time of 480 s, which is believed to be due to more severe spatial heterogeneity. Meanwhile, compared to CSPEs with 10, 15, and 20 wt % LIC active filler, the PEO-LiTFSI-30 wt % LIC sample possesses more electron-rich Li–O species after lithium evaporation times of 240 s, 360 s, and 600 s, while there are more electron-rich Li–S species after lithium evaporation times of 120 and 240 s, indicating that the increase in LIC content favors the formation of electron-rich Li–O and Li–S species.

**Table 1:** Atomic Concentration (At %) of Electron-Rich Li–O Species Derived from O 1s Core Levels (*i.e.*, 100% for Each Core Level) of Samples with Different Active Fillers under Pristine, During Stepwise Lithium Evaporation, and Post-HT Conditions

	*P*	120 s	240 s	360 s	480 s	600 s	HT
10 wt % LIC	-	11.3	3.1	4.8	29.7	11.2	-
15 wt % LIC	-	-	-	4.7	3.7	3.1	-
20 wt % LIC	-	-	6.3	2.2	50.2	2.6	6.4
30 wt % LIC	-	-	7.6	8.5	11.7	15.7	-
10 wt % LLZTO	-	-	-	2.0	-	2.3	-
10 wt % LPSC	-	-	1.1	0.8	3.3	4.6	-

**Table 2:** Atomic Concentration (At %) of Electron-Rich Li–S Species Derived from S 2p Core Levels (*i.e.*, 100% for Each Core Level) of Samples with Different Active Fillers under Pristine, During Stepwise Lithium Evaporation, and Post-HT Conditions

	*P*	120 s	240 s	360 s	480 s	600 s	HT
10 wt % LIC	4.1	4.9	4.5	4.4	20.2	6.6	2.9
15 wt % LIC	9.8	-	-	4.7	7.4	9.1	-
20 wt % LIC	-	-	11.1	16.7	46.1	16.5	5.9
30 wt % LIC	-	12.3	11.3	12.6	17.9	14.8	5.4
10 wt % LLZTO	-	-	-	-	-	-	-
10 wt % LPSC	6.8	4.3	3.7	4.0	5.3	4.4	16.6

We believe that the incorporation of LLZTO and LPSC fillers yields essentially a mechanical mixture and does not promote the interfacial reactions between PEO-LiTFSI and lithium metal, while In^3+^ from the LIC filler can facilitate the formation of electron-rich Li–O and Li–S, constituting a passivating SEI at the interface and thus improving the stability of CSPE against reactive lithium metal.

To further test the stability of PEO-LiTFSI-LIC against lithium metal, impedance spectroscopy as a function of resting time was conducted on a Li/PEO-LiTFSI-10 wt % LIC/Li symmetrical cell. Figure 5 shows all collected impedance spectra with different resting times fitted with equivalent circuits consisting of RQ elements (R—resistance, Q—constant phase element (CPE)). The first three impedance spectra, collected after the Li/PEO-LiTFSI-10 wt % LIC/Li symmetrical cell was left to rest for 3, 6, and 9 h at room temperature, exhibit mainly bulk conduction at high-frequency range with relatively high α values of 0.87, 0.82, and 0.83 (see Supporting Information, Table S1), which can be attributed to the poor contact between Li/PEO-LiTFSI-10 wt % LIC and Li metal caused by rough surface. After the symmetric cell was left to rest for longer time intervals, two more impedance spectra at 24 and 48 h were collected. We found that the impedance decreased significantly by 2 orders of magnitude, which is beneficial for increasing the critical current density for lithium dendrite formation. The processes at intermediate frequency ranges with lower α values of 0.42 and 0.41 (see Supporting Information, Table S2) are assigned as passivating SEI mainly containing electron-rich Li–O and Li–S species forming gradually upon contact between the Li/PEO-LiTFSI-10 wt % LIC and Li metal. According to X-ray photoelectron spectra of O 1s and S 2p during stepwise lithium metal evaporation, we believe a large amount of electron-rich Li–O and Li–S species can only form after a certain period of time.

**Figure 5:**
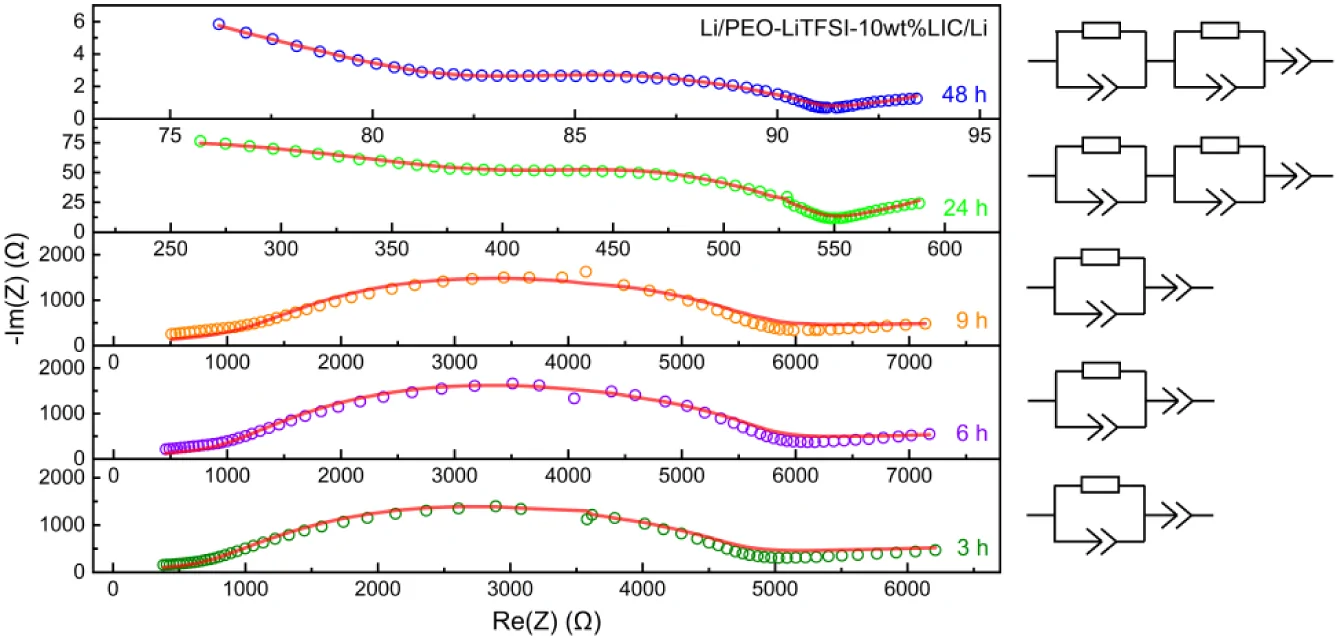
Resting time-dependent impedance spectra at different intervals of a symmetric Li/PEO-LiTFSI-10 wt % LIC/Li cell.

The Li/PEO-LiTFSI-10 wt % LIC/Li symmetrical cell can be stably cycled at 0.25 mAh cm^–2^ for 500 h (Figure 6a), indicating a stable stripping/plating behavior. In comparison, the Li/PEO-LiTFSI-10 wt % LLZTO/Li and Li/PEO-LiTFSI-10 wt % LPSC/Li symmetrical cells can only be cycled for short periods of time until short-circuited (Figure 6b and 6c), suggesting that LLZTO and LPSC as fillers are unfavorable for long-term cycling.

**Figure 6:**
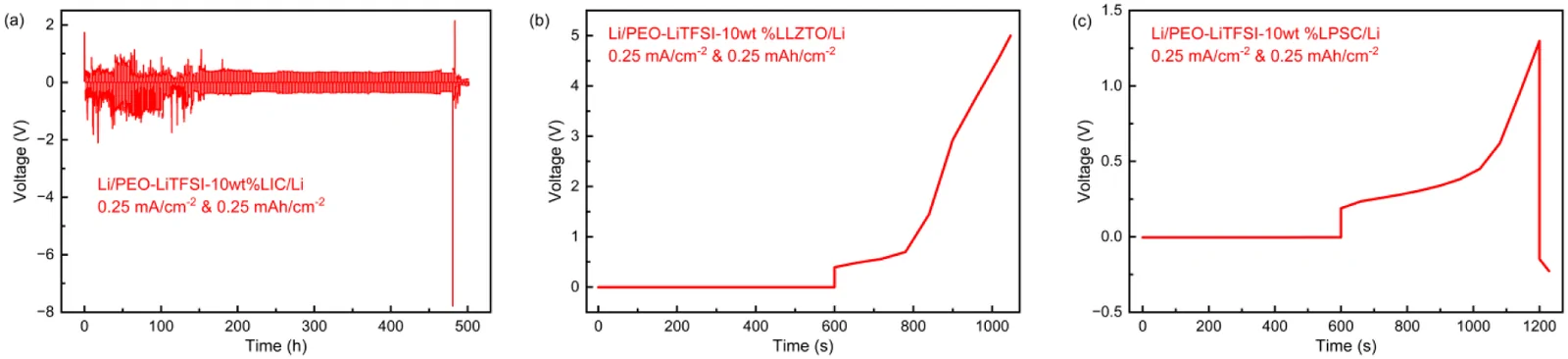
Cycling at 0.25 mAh cm^–2^ of (a) Li/PEO-LiTFSI-10 wt % LIC/Li, (b) Li/PEO-LiTFSI-10 wt % LLZTO/Li, and (c) Li/PEO-LiTFSI-10 wt % LPSC/Li symmetrical cells.

## Conclusions

Interface properties between a composite solid polymer electrolyte (CSPE) with halide- (Li_3_InCl_6_, LIC), oxide- (Li_6.4_La_3_Zr_1.4_Ta_0.6_O_12_, LLZTO), and sulfide- (Li_6_PS_5_Cl, LPSC)-based SE fillers and lithium metal are investigated via an *in-system* XPS analysis approach. First, according to XPS results, during stepwise lithium metal evaporation, PEO-LiTFSI-LIC samples can form large amounts of electron-rich Li–O and Li–S species acting as passivation layers. These species and metallic indium, induced by evaporated lithium metal, can be removed by low-temperature heat treatment to 90 °C for 3 h in an Ar atmosphere, viz., no further decomposition occurs by reduction reactions. Second, organic PEO and LiTFSI can absorb a certain amount of the evaporated lithium metal, thus reducing its negative impact on the stability of the CSPE. Third, PEO and LiTFSI themselves are able to form electron-rich Li–O and Li–S species with lithium metal, but only in small amounts. For oxide-, sulfide-, and halide-based SE fillers, the addition of LLZTO almost shows no effect, while LPSC has a counter effect, resulting in even more electron-rich Li–S species after HT. In contrast, incorporation of LIC can facilitate the formation of electron-rich Li–O and Li–S species acting as a passivation layer, thus improving the stability of CSPE against lithium metal. Finally, resting-time-dependent impedance spectroscopy characterization shows that the incorporation of LIC filler reduces the CSPE/Li interfacial impedance, which is beneficial for increasing the critical current density for lithium dendrite formation. Symmetric cells with lithium metal electrodes could be cycled for over 500 h with a stable stripping/plating behavior. The incorporation of halide-based filler into solid polymer electrolytes to induce passivating interphases improves the interfacial stability against the lithium metal anode and thereby offers a novel strategy for interfacial engineering of all-solid-state lithium metal batteries.

## Supplementary Material



## Data Availability

Research data supporting the figures and tables in the main manuscript and supporting information are made available under Zenodo.org via https://doi.org/10.5281/zenodo.21828814.

## Notes

X-ray photoelectron spectra of In 3d under different conditions and XRD measurements of four CSPE samples with different LIC content; X-ray photoelectron spectra of O 1s of CSPE samples with LIC contents of 10%, 15%, and 20%, respectively, at pristine, during stepwise lithium evaporation, and after heat treatment conditions; X-ray photoelectron spectra of S 2p of CSPE samples with LIC contents of 10%, 15%, and 20%, respectively, at pristine, during stepwise lithium evaporation, and after heat treatment conditions; X-ray photoelectron spectra of Li 1s of CSPE samples with LIC contents of 10%, 15%, and 20%, respectively, at pristine, during stepwise lithium evaporation, and after heat treatment conditions; X-ray photoelectron spectrum of O 1s of pure PEO sample under pristine, during stepwise lithium evaporation, and after heat treatment conditions; X-ray photoelectron spectra of O 1s and S 2p of PEO-LiTFSI sample under pristine, during stepwise lithium evaporation, and after heat treatment conditions; SEM images of PEO-LiTFSI-30 wt % LIC sample after stepwise lithium evaporation and low-temperature HT in magnifications of 250×, 3500×, and 10,000×; fitting parameters of equivalent circuits (Q1/R1+Q2) for impedance spectra of a Li/PEO-LiTFSI-10 wt % LIC/Li symmetrical cell collected with resting times of 3, 6, and 9 h at room temperature; fitting parameters of equivalent circuits (Q1/R1+Q2/R2+Q3) for impedance spectra of a Li/PEO-LiTFSI-10 wt % LIC/Li symmetrical cell collected with resting times of 24 and 48 h at room temperature; schematic diagrams of stepwise lithium evaporation onto samples and DArmstadt Integrated SYstem for BATtery Research (DAISY-BAT) (PDF)
