# Supplementary material for: Li3InCl6 as an Active Filler Improves Interfacial Stability of PEO-LiTFSI Composite Solid Polymer Electrolytes for All-Solid-State Lithium Metal Batteries
Source: ACS Omega. 2026 Aug 20;11(34):50883–91. doi: 10.1021/acsomega.6c02071 (PMC13625121; doi:10.1021/acsomega.6c02071)
Supplement: Supplementary file 1 [file ao6c02071_si_001.pdf]

Supporting Information

Li_3InCl_6 as an Active Filler Improves Interfacial Stability of PEO-LiTFSI Composite Solid Polymer Electrolytes for All-Solid-State Lithium Metal Batteries

Hongrui Kang[a], Weiye Ma[b], Jan P. Hofmann[a,*] and Zhonghui Gao[b,*]

[a] Surface Science Laboratory, Department of Materials- and Geosciences, Peter-Grünberg-Straße 4, Technical University of Darmstadt, 64287 Darmstadt, Germany

[b] School of Materials Science and Engineering, Tianjin University, Tianjin 300350, P. R. China

*Corresponding authors

Email: zhgao@tju.edu.cn

Email: hofmann@surface.tu-darmstadt.de

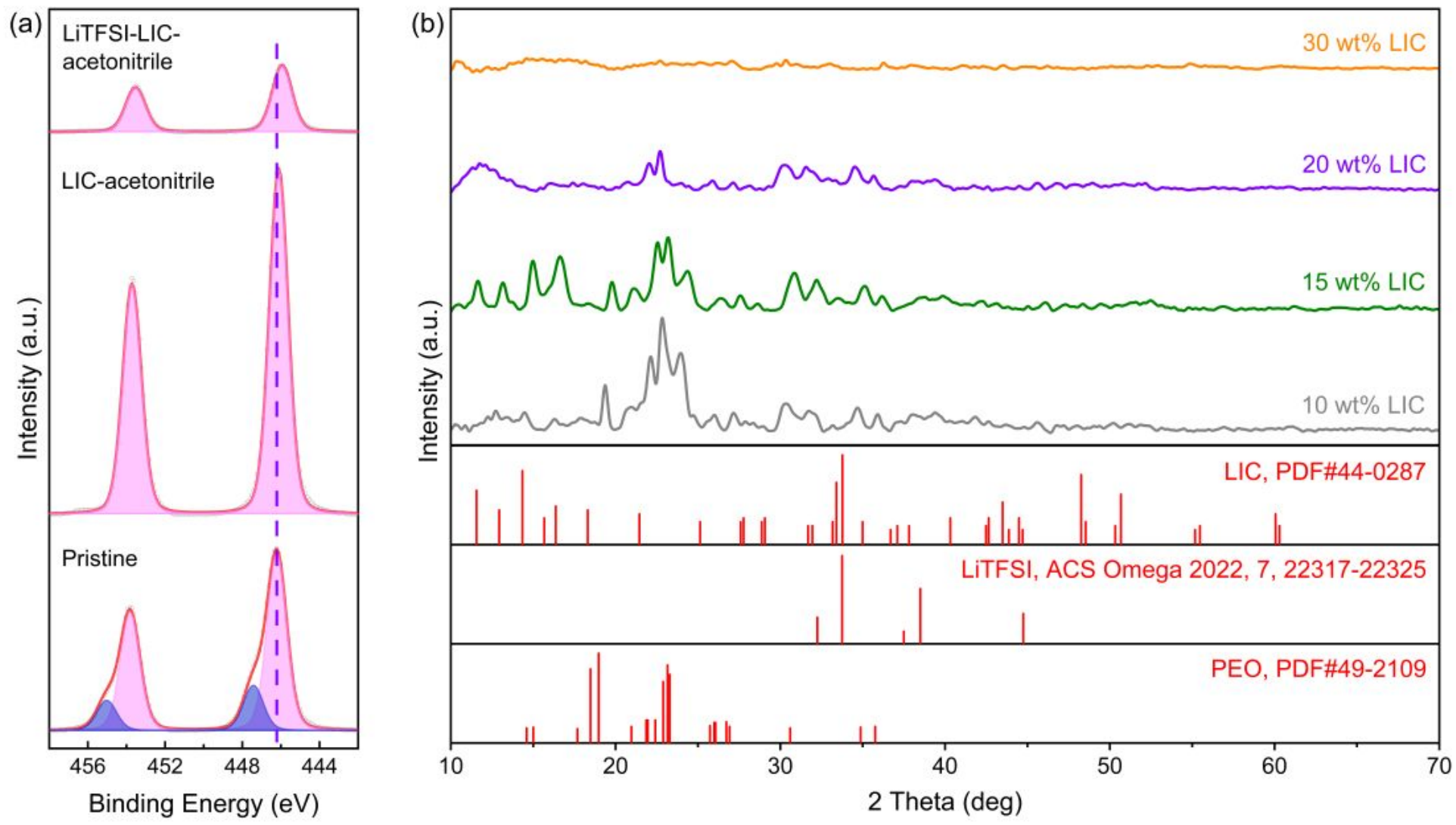

Figure S1. (a) X-ray photoelectron spectra of In 3d under different conditions; (b) X-ray diffractogram of CSPEs with different LIC content as well as reference samples, as the LIC content increases, the degree of amorphization increases.

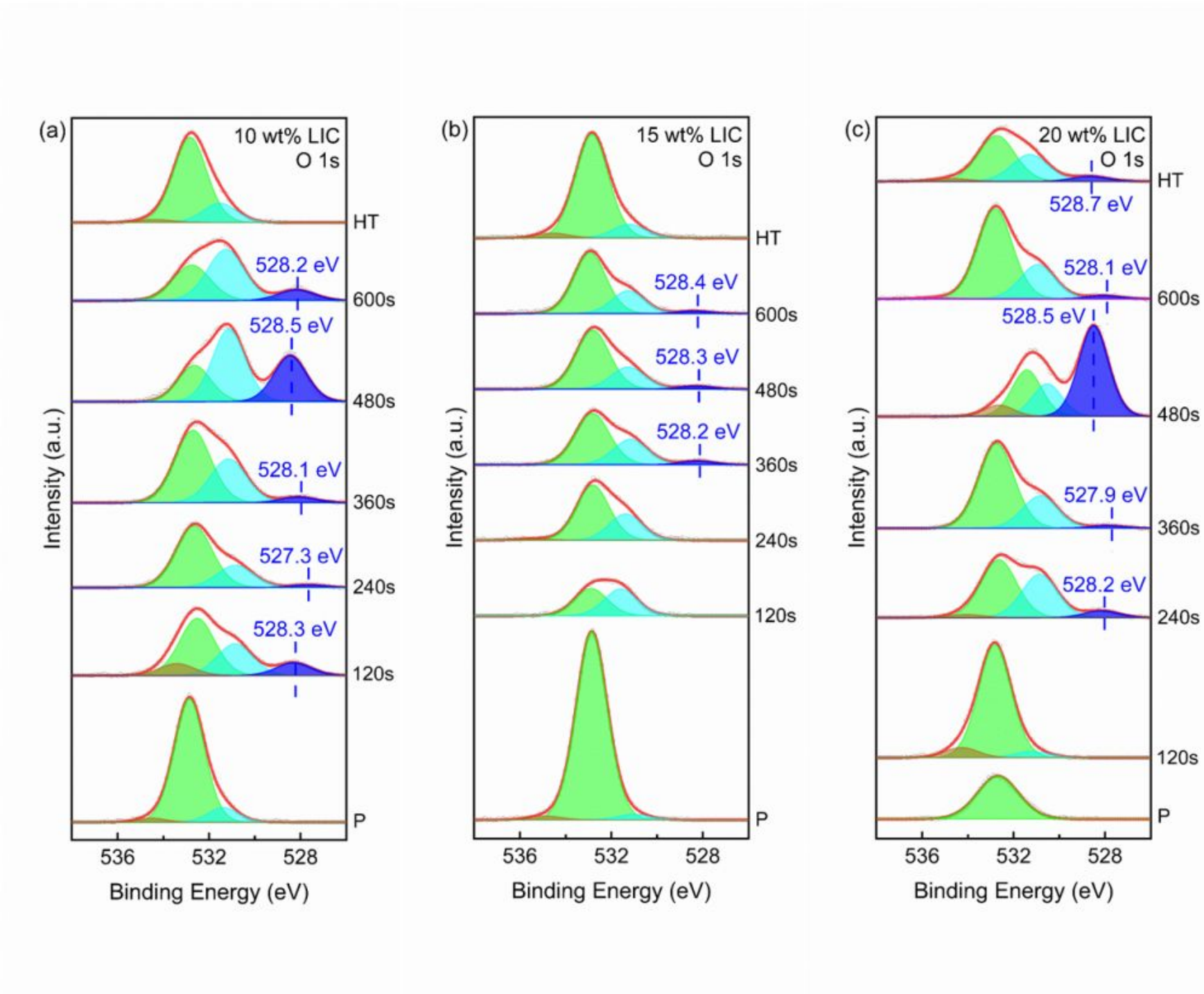

Figure S2. X-ray photoelectron spectra of O 1s of (a) PEO-LiTFSI-10wt %LIC[1], (b) PEO-LiTFSI-15wt %LIC, and (c) PEO-LiTFSI-20wt %LIC samples at pristine, during stepwise lithium evaporation, and post HT conditions.

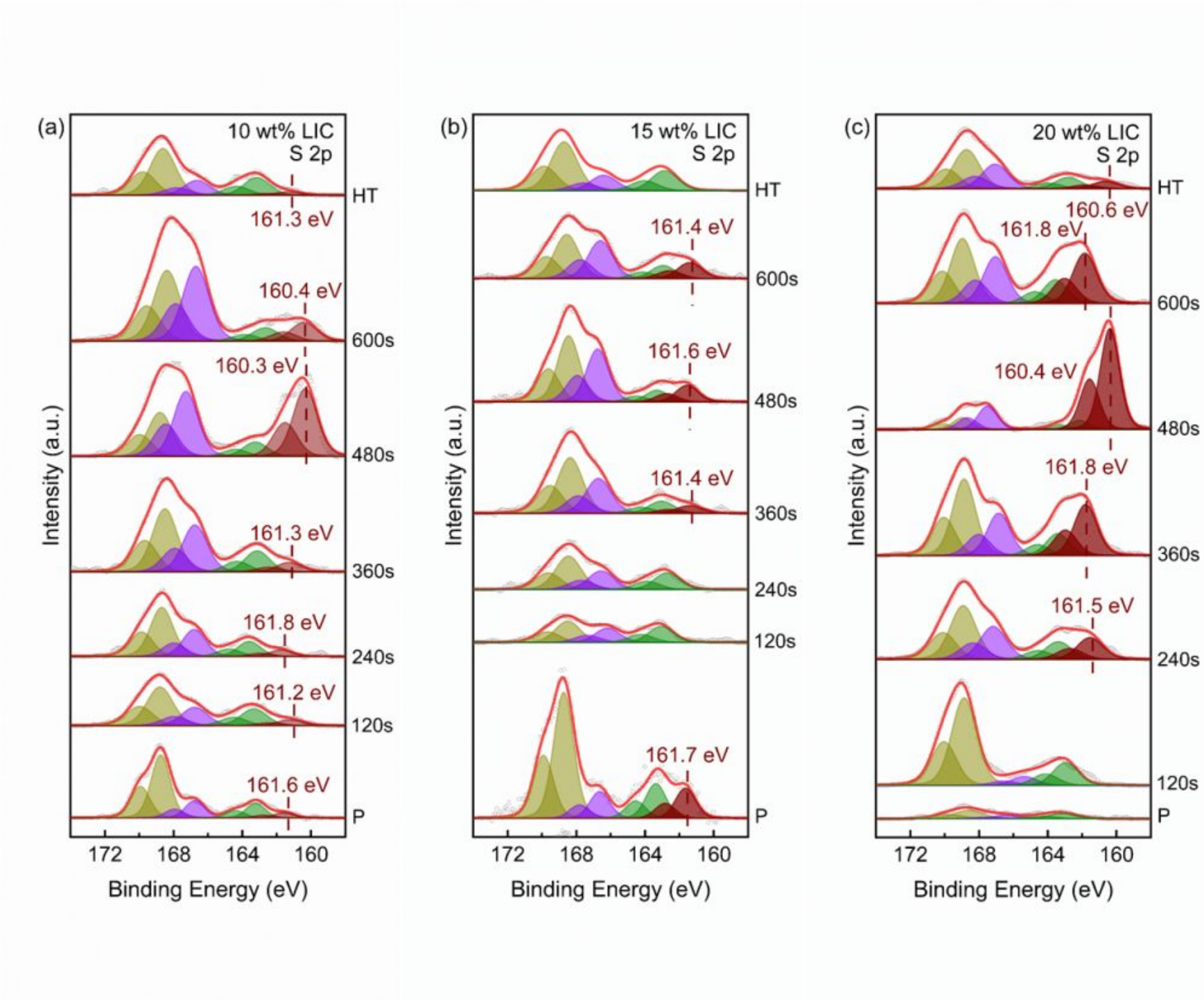

Figure S3. X-ray photoelectron spectra of S 2p of (a) PEO-LiTFSI-10wt %LIC[1], (b) PEO-LiTFSI-15wt %LIC, and (c) PEO-LiTFSI-20wt %LIC samples at pristine, during stepwise lithium evaporation, and post HT conditions.

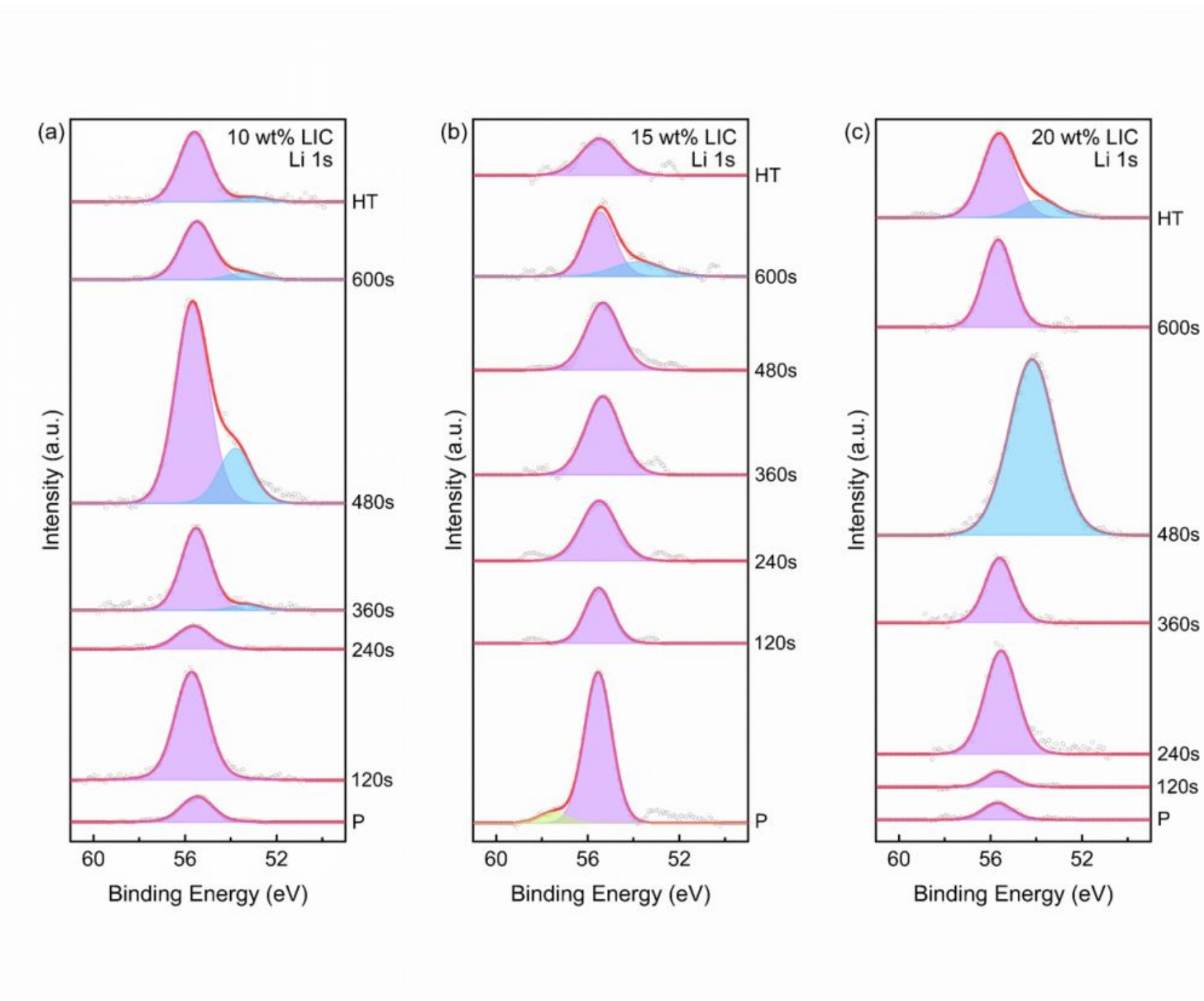

Figure S4. X-ray photoelectron spectra of Li 1s of (a) PEO-LiTFSI-10wt %LIC[1], (b) PEO-LiTFSI-15wt %LIC, and (c) PEO-LiTFSI-20wt %LIC samples at pristine, during stepwise lithium evaporation, and post HT conditions.

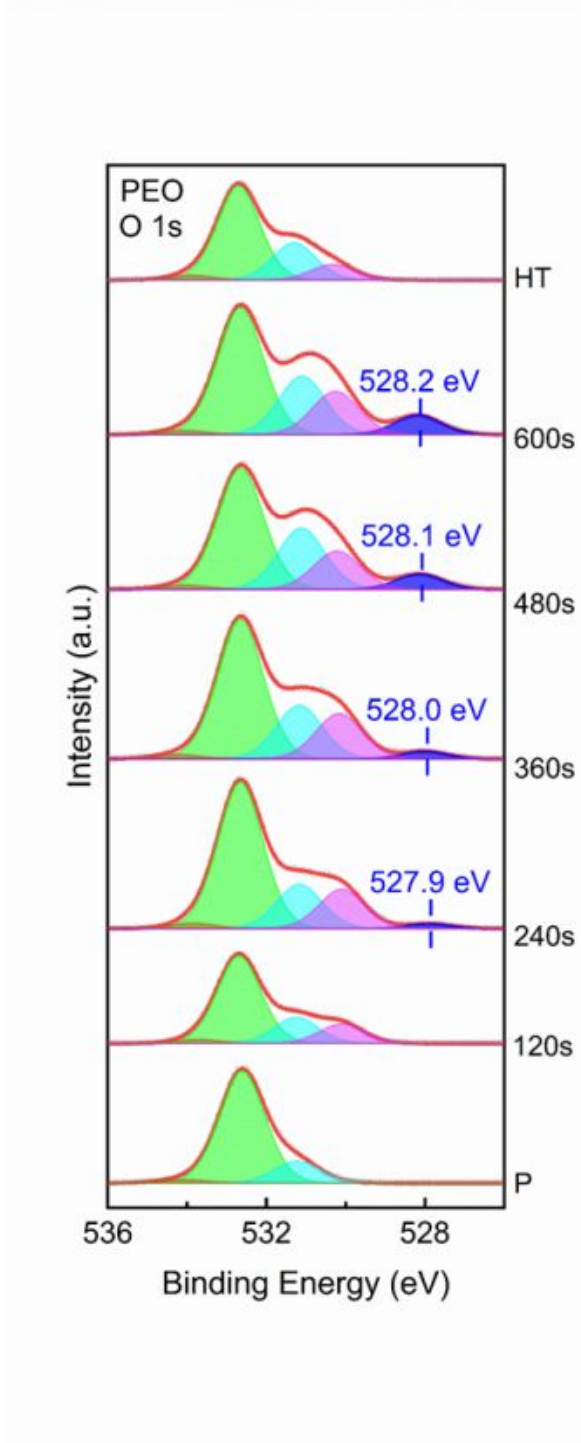

Figure S5. X-ray photoelectron spectra of O 1s of pure PEO sample at pristine, during stepwise lithium evaporation, and after heat treatment conditions.

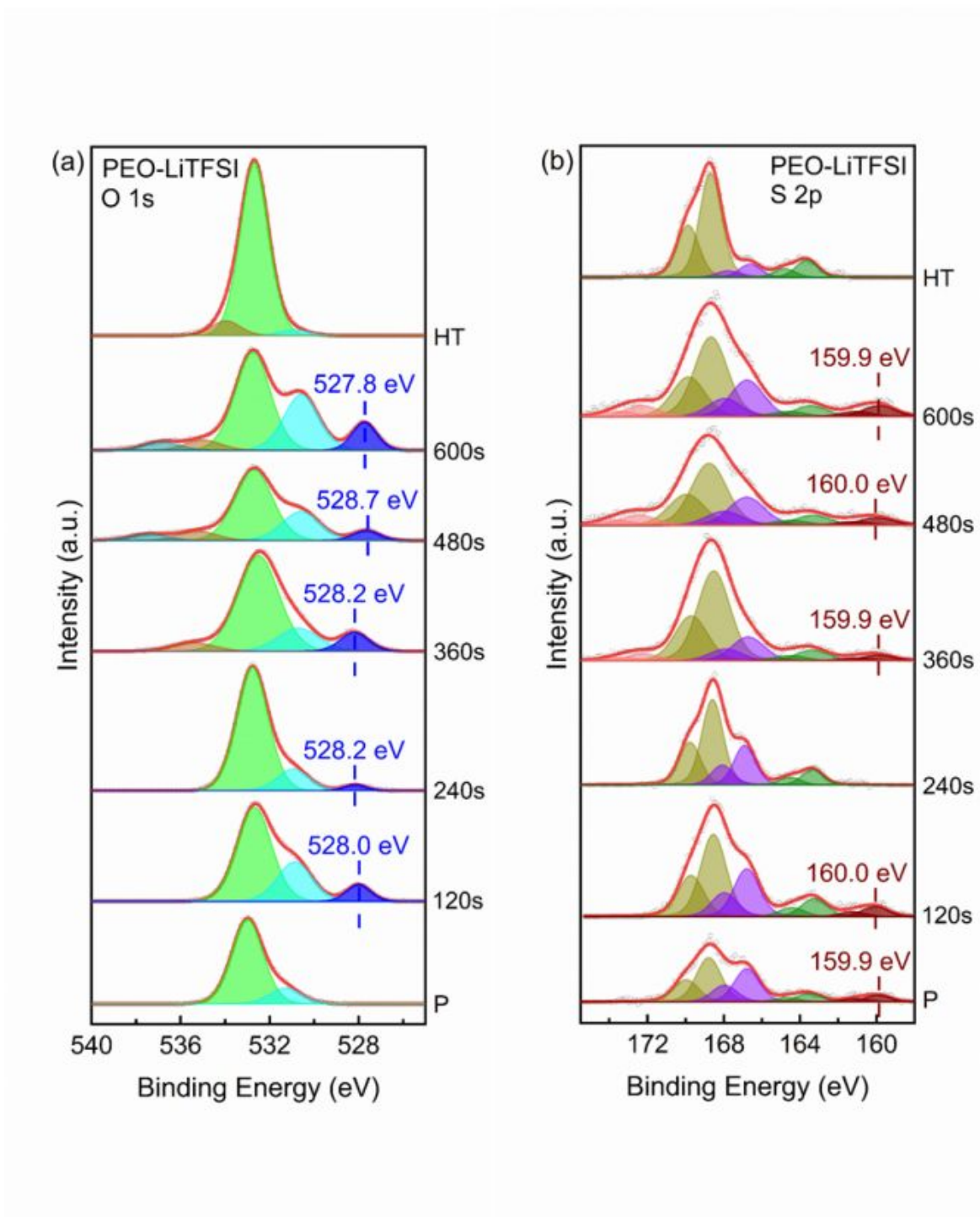

Figure S6. X-ray photoelectron spectra of (a) O 1s and (b) S 2p of PEO-LiTFSI sample at pristine, during stepwise lithium evaporation, and post HT conditions.

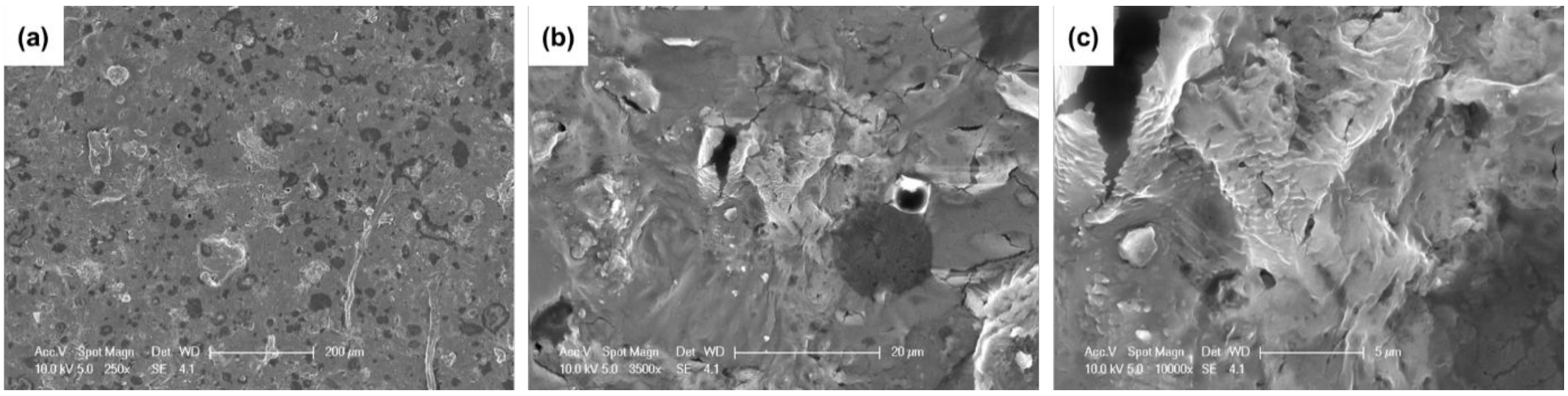

Figure S7. SEM images of PEO-LiTFSI-30wt %LIC sample after stepwise lithium evaporation and low-temperature heat treatment in different magnifications: (a) 250x, (b) 3500x, and (c) 10000x.

Table S1. Fitting parameters of equivalent circuits (Q1/R1+Q2) for impedance spectra of a Li/PEO-LiTFSI-10wt %LIC/Li symmetrical cell collected with the resting time of 3 h, 6 h, and 9 h at room temperature.

	Q1 (Fs^{a-1})	α1	R1 (Ω)	Q2 (Fs^{a-1})	α2
3 h	(1.58 ± 0.14)E−6	0.87 ± 0.02	(2.71 ± 0.06)E3	(0.47 ± 0.02)E−3	(1.40 ± 0.03)E−1
6 h	(1.60 ± 0.07)E−6	0.82 ± 0.01	(3.70 ± 0.06)E3	(0.46 ± 0.01)E−3	(1.50 ± 0.04)E−1
9 h	(1.65 ± 0.12)E−6	0.83 ± 0.01	(3.25 ± 0.04)E3	(0.46 ± 0.01)E−3	(1.40 ± 0.03)E−1

Table S2. Fitting parameters of equivalent circuits (Q1/R1+Q2/R2+Q3) for impedance spectra of a Li/PEO-LiTFSI-10wt %LIC/Li symmetrical cell collected with the resting time of 24 h and 48 h at room temperature.

	Q1 (Fs^{a-1})	α1	R1 (Ω)	Q2 (Fs^{a-1})	α2	R2 (Ω)	Q3 (Fs^{a-1})	α3
24 h	(39.19 ± 0.87)E−6	0.70 ± 1.74E−3	(1.06 ± 0.01)E2	(5.60 ± 32.86)E−6	0.42 ± 0.53E−3	(4.36 ± 0.01)E2	0.02 ± 0.30E−3	0.33 ± 2.17E−3
48 h	(0.43 ± 0.02)E−3	0.67 ± 3.59E−3	13.95 ± 0.17	(3.88 ± 49.01)E−6	0.41 ± 0.89E−3	(16.96 ± 0.02)E1	0.09 ± 1.84E−3	0.25 ± 4.77E−3

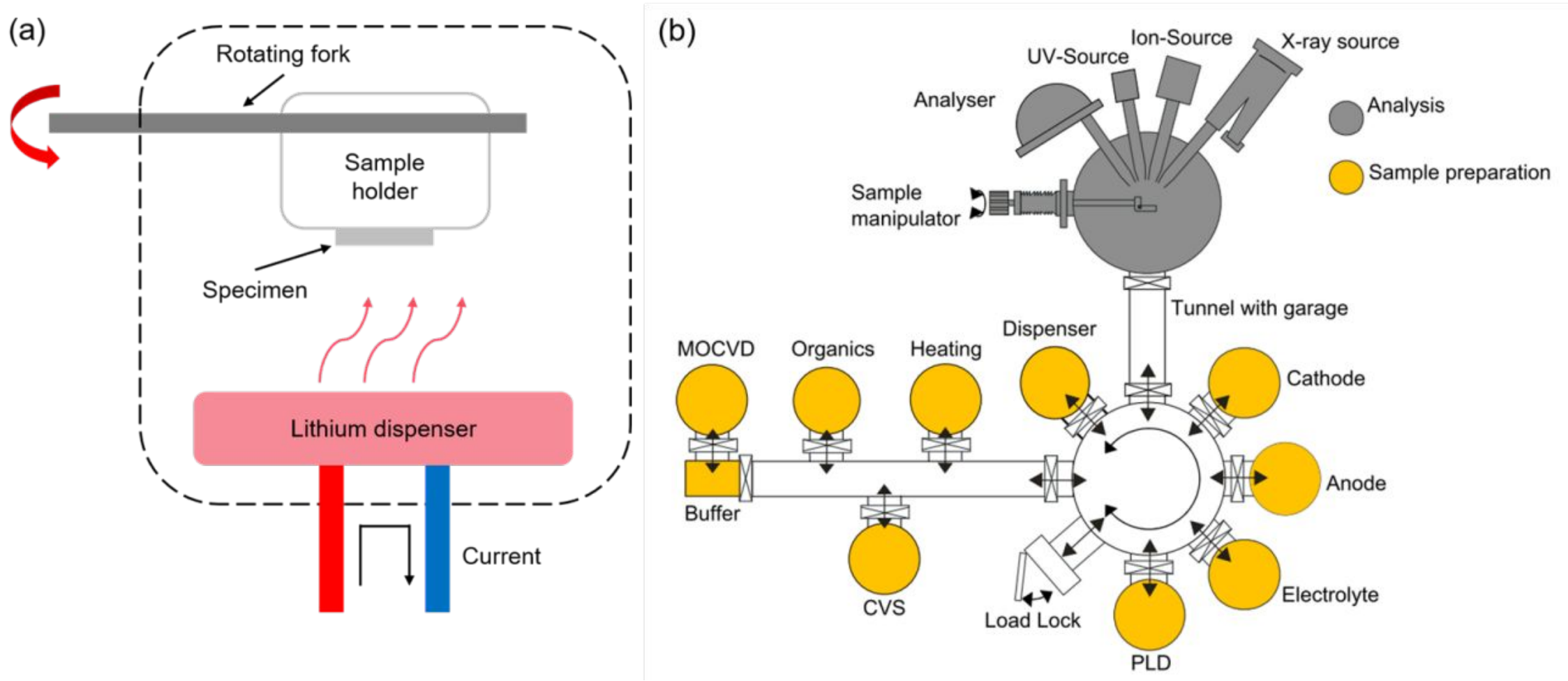

Figure S8. Schematic diagrams of (a) stepwise lithium evaporation onto samples; (b) DArmstadt Integrated SYstem for BATtery Research (DAISY-BAT)

References

(1) Ma, W.; Zhang, L.; Zhu, G.; Kang, H.; Yuan, H.; Long, Y.; Pan, Z.; Liu, Z.; Liu, X.; Liang, J.; Liang, Y.; Cui, Z.; Zhu, S.; Zhao, Y.; Gao, Z. Acetonitrile-Driven Structural Change of Li_3InCl_6 Enables Amorphization of PEO-LiTFSI for Enhanced Room-Temperature Performance. *J. Mater. Chem. A* **2026**, 10.1039.D6TA00729E. https://doi.org/10.1039/D6TA00729E.
